# Nifurtimox versus benznidazole or placebo for asymptomatic *Trypanosoma cruzi* infection (Equivalence of Usual Interventions for Trypanosomiasis - EQUITY): study protocol for a randomised controlled trial

**DOI:** 10.1186/s13063-019-3423-3

**Published:** 2019-07-15

**Authors:** Juan Carlos Villar, Víctor Mauricio Herrera, Juan Guillermo Pérez Carreño, Eliana Váquiro Herrera, Yeny Zulay Castellanos Domínguez, Skarlet Marcell Vásquez, Zulma Milena Cucunubá, Nilda Graciela Prado, Yolanda Hernández

**Affiliations:** 1 Grupo de Cardiología Preventiva, Facultad de Ciencias de la Salud, Universidad Autónoma de Bucaramanga, Calle 157 No 19- 55, Campus el Bosque, Bucaramanga, Colombia; 2 Departamento de Investigaciones, Fundación Cardioinfantil- Instituto de Cardiología, Bogotá, Colombia; 3 Dirección de Investigación e Innovación, Universidad del Rosario, Bogotá, Colombia; 4 Grupo de Parasitología, Instituto Nacional de Salud, Bogotá,, Colombia; 5 MRC Centre for Global Infectious Disease Analysis (MRC GIDA), Department of Infection Disease Epidemiology, Imperial College London, London, UK; 6 Departamento de Clínica, Patología y Tratamiento, Instituto Nacional de Parasitología Mario Fatala Chabén, Buenos Aires, Argentina

**Keywords:** Nifurtimox, Benznidazole, Trypanosoma cruzi, Chagas disese, Randomized controlled trial

## Abstract

**Background:**

Either benznidazole (BZN) or nifurtimox (NFX) is recommended as equivalent to treat *Trypanosoma cruzi* infection. Nonetheless, supportive data from randomised trials is limited to individuals treated with BZN in southern cone countries of Latin America.

**Methods:**

The goal of this randomised, concealed, blind, parallel-group trial is to inform the trypanocidal efficacy and safety of NFX and its equivalence to BZN among individuals with *T. cruzi* positive serology (TC+). Eligible individuals are TC+, 20–65 years old, with no apparent symptoms/signs or uncontrolled risk factors for cardiomyopathy and at negligible risk of re-infection. Consenting individuals (adherent to a 10-day placebo run-in phase) receive a 120-day BID blinded treatment with NFX, BZN or matching placebo (2:2:1 ratio). The four active medication arms include (1) a randomly allocated sequence of 60-day, conventional-dose (60CD) regimes (BZN 300 mg/day or NFX 480 mg/day, ratio 1:1), followed or preceded by a 60-day placebo treatment, or (2) 120-day half-dose (120HD) regimes (BZN 150 mg/day or NFX 240 mg/day, ratio 1:1). The primary efficacy outcome is the proportion of participants testing positive at least once for up to three polymerase chain reaction (PCR) assays (1 + PCR) 12–18 months after randomisation. A composite safety outcome includes moderate to severe adverse reactions, consistent blood marker abnormalities or treatment abandons. The trial outside Colombia (expected to recruit at least 60% of participants) is pragmatic; it may be open-label and not include all treatment groups, but it must adhere to the randomisation and data administration system and guarantee a blinded efficacy outcome evaluation. Our main comparisons include NFX groups with placebo (for superiority), NFX versus BZN groups and 60CD versus 120HD groups (for non-inferiority) and testing for the agent-dose and group-region interactions. Assuming a 1 + PCR ≥ 75% in the placebo group, up to 25% among BZN-treated and an absolute difference of up to ≥ 25% with NFX to claim its trypanocidal effect, 60–80 participants per group (at least 300 from Colombia) are needed to test our hypotheses (80–90% power; one-sided alpha level 1%).

**Discussion:**

The EQUITY trial will inform the trypanocidal effect and equivalence of nitroderivative agents NFX and BZN, particularly outside southern cone countries. Its results may challenge current recommendations and inform choices for these agents.

**Trial registration:**

ClinicalTrials.gov, NCT02369978. Registered on 24 February 2015.

**Electronic supplementary material:**

The online version of this article (10.1186/s13063-019-3423-3) contains supplementary material, which is available to authorized users.

## Background

The World Health Organization (WHO) estimates that 8 million people are infected by *Trypanosoma cruzi*, the parasite causing Chagas disease, and 10,000 of these people are expected to die every year [1]. Different health authorities recommend either nifurtimox (NFX) or benznidazole (BZN) as treatment for *T. cruzi*-infected individuals [2–5]. The purpose of using these trypanocidal agents is to reduce the parasitic load (expecting to prevent, delay or reduce the impact of clinical complications) and to reduce its transmission [6]. However, a number of reasons hinder this potential opportunity. Firstly, convincing evidence of efficacy from randomised trials is limited to parasite-related (but not patient-important) outcomes [7], to treatment with BZN (but not NFX) and to individuals from Brazil and Argentina and more recently Bolivia (but not other countries in Latin America) [8–13]. Secondly, the tolerance to this treatment, typically offered for 60–90 days, is still suboptimal, with an abandon rate ranging from10 to 20% reported in these studies.

In the above context, decision-makers, at both the individual and population levels, will benefit from more information on the efficacy and safety of NFX as treatment for *T. cruzi* infection. The trypanocidal effect of NFX, demonstrated in a single trial in Brazil [14], needs replication and validation in other populations. Furthermore, treatment with BZN in the BENEFIT trial proved to be trypanocidal for participants from Argentina and Brazil (80% of the study population) but not for (the remaining 20%, 502 and 78) participants from Colombia and El Salvador [11]. Geographical variability of trypanocidal efficacy of BZN had been previously reported in a large case series of school children from Bolivia, Honduras and Guatemala [15]. This highlights the importance of expanding the existing information on the trypanocidal efficacy of NFX and exploring its equivalence to BZN. This emerging information may be of particular interest for endemic countries with smaller representation in previous trials, such as Colombia.

In the direction of addressing this research gap, this paper describes the methods for Phase 3 of the Cardiovascular Health Investigation and Collaboration from Countries of America to Assess the Markers and Outcomes of Chagas disease (CHICAMOCHA 3) project, the Equivalence of Usual Interventions for Trypanosomiasis (EQUITY) trial.

## Methods

Overall, the aim of the EQUITY trial is to evaluate, among young adults with chronic *T. cruzi* infection but no symptomatic chronic Chagas cardiomyopathy, the trypanocidal effect and safety of a treatment with NFX compared with BZN or placebo.

### Study design

We will conduct a randomised, concealed, multi-centre, parallel-group, blind trial, testing the superiority of NFX over placebo and its non-inferiority compared with BZN.

### Setting and participants

EQUITY will seek collaborating centres from Colombia and other countries in Latin America offering care to outpatients with *T. cruzi* chronic infection. Study centres will be actively seeking eligible candidates with local centres for serology diagnosis, blood banks or health authorities. The eligible population will be individuals aged 20–65 of both sexes, with serology diagnosis (positive results for at least two out of three serological tests including enzyme-linked immunosorbent assay [ELISA] and indirect immunofluorescence of haemagglutination over the last 10 years), negligible risk of re-infection (residence in an urban setting or a suburban/rural area with no history of vector infestation or transmission) and the capability to attend regular follow-up visits at the study centres (Table 1).

**Table 1:** Study eligibility criteria

Eligibility	Exclusion criteria
• Men or women 20–65 years of age	• Previous treatment with nifurtimox or benznidazole
• Positive serology for *T. cruzi* (at least two different techniques applied to the same blood sample) in the last 10 years	• Lack of compliance/tolerance to a 10-day run-in period
• No clinical suspicion, or active risk factors for, chronic cardiomyopathy	• Abnormal values in blood cell counts or laboratory tests for hepatic and renal function
• Negligible risk of vector *T. cruzi* re-infection	• Any medical/social condition hindering free participation
• Capable of complying with the planned study visits	• Current or planned pregnancy, or not using secure birth methods for women of childbearing age

Exclusion criteria include the following: previous treatment with BZN or NFX or participation in trials testing trypanocidal drug candidates; any symptom suggesting chronic Chagas (or other forms) of cardiomyopathy or uncontrolled risk factors for it; persistent abnormalities in liver/kidney blood function tests (alanine amino transferase [ALAT]/aspartate amino transferase [ASAT] twofold normal values or creatinine over 1.2 mg/dl observed at least twice); women of childbearing age who have positive pregnancy tests or are unwilling to use secure birth control methods; or any concomitant health problem judged by the study physician as potentially interfering with free participation, health or well-being during the study or administration of treatment.

### Interventions

The randomly allocated study interventions may differ for centres across countries. While in Colombia EQUITY will offer masked (i.e. similar-looking) treatments, including placebo or active treatments, in the remaining centres it will be a pragmatic, open-label trial, where the control group may not receive medication (see further explanation in the following paragraphs).

In Colombian centres, before randomisation, in order to ensure adherence to study interventions, eligible, consenting individuals will go over a 10-day, run-in treatment (with placebo). This treatment placebo, blinded for participants, has a similar appearance to the study drug and will be given in similar fashion (2 capsules twice a day [BID]). Those reporting tolerance and adherence (not experiencing symptoms judged as moderate/severe and taking at least 80% of the capsules) will be prescribed a blinded treatment of 2 capsules BID for 120 days. This includes a sequence-concealed, randomly allocated treatment with any of the following five treatment groups (ratio 1:1:1:1:1):

- NFX 480 mg/day for 60 days
- NFX 240 mg/day for 120 days
- BZN 300 mg/day for 60 days
- BZN 150 mg/day for 120 days
- Placebo

**Fig. 1:**
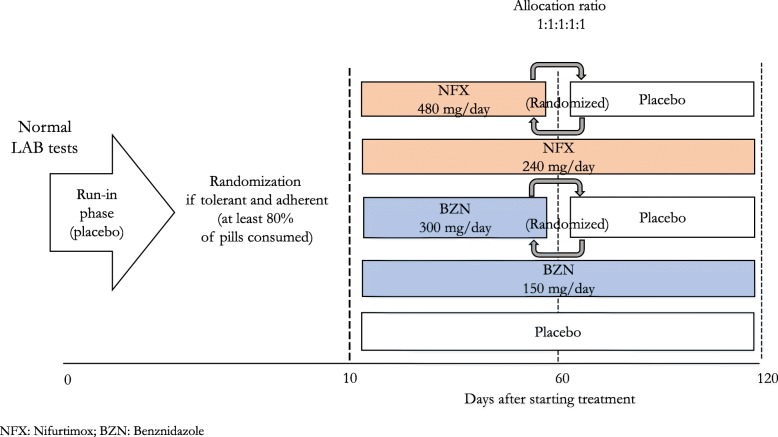
Process of treatment allocation

**Fig. 2:**
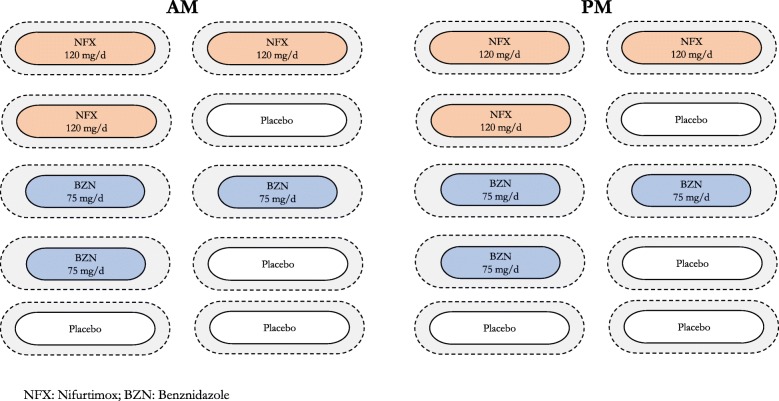
Daily 60-day treatment schedule for study participants

Two of the four groups with active treatment will have a 60-day, conventional dose (60CD) of either NFX or BZN, whereas the other two groups include 120-day, half-dose (120HD) treatment. As the prescribed study treatment for all groups takes 120 days, the 60CD groups will receive another 60-day masked placebo treatment that precedes or follows the active treatment in a randomly assigned sequence. Figure 1 summarises the study arms and assigned treatments. To ensure the blinding to participants and their relatives, treating physicians and study teams, the study medication will be packaged in a similar fashion. We used similar gelatin capsules to include and mask the contents of the original tablets of active treatments or placebo (a mix of microcrystalline cellulose and magnesium stearate), as shown in Fig. 2. The study medication was packed in bottles of 120 capsules (intended to last for 2 months) labelled with the study name and treatment codes, making the study arms indistinguishable from each other.

Outside Colombia, because of (1) resource constraints to organise a centralised shipment of study medication or to mask locally their study medication, or (2) local interpretation of the evidence, clinical practice patterns and/or regulations on using trypanocidal treatments, the protocol may have variations in the interventions. EQUITY investigators at those centres will have flexibility to decide on (1) which of the proposed active treatment groups to include and (2) whether or not to have a control group receiving no treatment. Groups receiving active treatment may be any two or all four treatment groups described above. If two groups are chosen, they must use either different agents given at the same dose level and treatment length (either 60CD or 120HD) or the same agent (either NFX or BZN) given at different dose levels and treatment lengths. The choice of including a group not receiving active treatment will depend on the judgement of local investigators or regulatory agencies on whether it is necessary (1) to prove a trypanocidal effect of NFX or (2) to start trypanocidal treatment right upon serology diagnosis.

For centres outside Colombia choosing to include a group receiving no treatment, the allocation ratio will be the same for each active treatment group (i.e. 1:1:1 for those having two active treatment groups and 1:1:1:1:1 for those including all four active treatment groups). In all circumstances, however, all EQUITY centres will use a computerised central randomisation system with a concealed sequence to allocate the local choice of study treatments. All study sites will also have to comply with a blinded process for outcome assessment.

### Comparisons

EQUITY will test two main efficacy hypotheses, driving the primary comparisons. Firstly, we will test the superiority of NFX, comparing a composite of groups receiving this medication against those allocated to placebo/no treatment (trypanocidal effect of NFX). Secondly, we will test the non-inferiority of (1) a composite of NFX groups compared with those including BZN and (2) 60CD regime groups of any active medication compared with those receiving 120HD regimes. The above comparisons place BZN/60CD as standard treatment, assuming its superiority to placebo. Under this hypothesis, for NFX/120HD to be a valid alternative, we will require its trypanocidal efficacy to be at least two thirds of that for BZN/60CD (see the statistical assumptions made in the “Sample size calculations” section).

Centres outside Colombia may provide data for some, but not all, planned comparisons, depending on their choice of treatment groups. For example, data from centres including only active treatment groups with both NFX and BZN will contribute to test the hypothesis of non-inferiority of NFX to BZN but not to evaluate the trypanocidal efficacy itself. Whatever the choice of treatment groups, however, under our study design our primary comparisons may vary in the number of participants included, but they will only be between randomly allocated treatment groups at a centre level.

We will also test the dose-agent and the treatment-region (southern/northern) interactions for the above comparisons. Data from countries outside Colombia will be included in the applicable comparisons, depending on their treatment groups. We plan to conduct separate sensitivity analyses for our primary comparisons including all randomised participants and those included in Colombia. The safety evaluation will follow the same comparisons described for efficacy.

Secondary comparisons are intended only for efficacy of BZN. They will include (1) testing its superiority over placebo, testing for the treatment-region interaction, and (2) testing the non-inferiority of BZN 120HD compared with BZN 60CD in the overall population. As for the primary comparisons, data from centres outside Colombia may be included, provided they have chosen to randomise their participants to each of these groups.

A subgroup, exploratory analysis will include (1) age strata at inclusion (20–44/45–65 years), (2) presence/absence of electrocardiographic abnormalities and (3) region of origin (southern/northern South America) for all the planned efficacy comparisons.

### Outcomes

#### Efficacy

The primary outcome will be the proportion of participants testing positive at least once for up to three independent assays of polymerase chain reaction (PCR) during the 12–18 months after the initiation of treatment, with an interval of 7 or more days from each other. Secondary efficacy outcomes will be (1) the mean change in the levels of B-type natriuretic peptides (either NT pro-BNP or BNP, as long as the same test is used for each participant) and (2) the proportion of participants with positive serological status (using conventional serology diagnosis) 12 months after the initiation of treatment. Centres outside Colombia may or may not, depending on the resources available and practice patterns, provide data on BNP or conventional serology after treatment. All efficacy outcomes will be recorded and analysed without knowledge of treatment allocation.

#### Safety

The primary safety outcome will be a composite proportion of participants meeting at least one of the following conditions:

1. Hospitalisations or medical leaves (as signed by physicians outside the study)
2. Interruption of treatment (by the study physician or participant’s initiative) for at least 30 days due to suspected side effects, or not taking the assigned treatment for at least 90 continuous days (75% of the treatment)
3. The incidence of sustained abnormalities (values twofold over cut-off) in at least two biochemical or blood markers monitored during the experimental treatment at least twice with an interval of minimum 2 weeks, up to 1 month after finishing the allocated treatment

Secondary outcomes will be

1. The incidence of signs (e.g. skin reactions) or symptoms (e.g. dyspepsia, headache, numbness or neuropathic pain) during treatment, considered moderate or severe (needing temporary or definitive suspension of treatment) by the study physician
2. Changes in biochemical and blood markers during the first month of treatment with respect to baseline.

Whenever possible, investigators from centres outside Colombia will make efforts to record safety outcome data blinded to the allocated treatment. As this may not be entirely possible, we will conduct a sensitivity analysis for safety outcomes at the Colombian centres and the entire study population separately.

### Study procedures and follow-up of participants

Once they have been screened for eligibility, offered participation in the study and have given their consent, candidates will start the run-in phase (visit 0). Study physicians will rule out any exclusion criteria for those completing this phase and, for those still eligible, will randomise a new participant (visit 1). The participants will receive the allocated treatment for the first 60 days and will be asked to attend a number of follow-up visits during treatment. These visits allow reporting of any potential side effects as well as providing physical exams and/or blood work safety monitoring. For Colombian centres, the visits are scheduled at days 20, 30 and 60, repeating the cycle when a second 60-day cycle starts.

In the presence of signs of intolerance, study physicians may decide to stop the study medication temporarily or definitely. They may also prescribe a symptomatic treatment (e.g. an anti-histaminic for pruritus) to participants over the study period or refer to treating physicians or medical emergency services when they judge it may be necessary. Adherence, as the reciprocal proportion of scheduled treatment that participants left unused, will be assessed at visits 2 through 7. Efficacy outcome data will be collected during months 12 to 18 after treatment start, scheduling three separate visits (with at least 1 week of separation) at each participant’s convenience. Table 2 summarises the study follow-up visits scheme. The Standard Protocol Items: Recommendations for Interventional Trials (SPIRIT) checklist is included as an additional file (see Additional file 1).

**Table 2:**
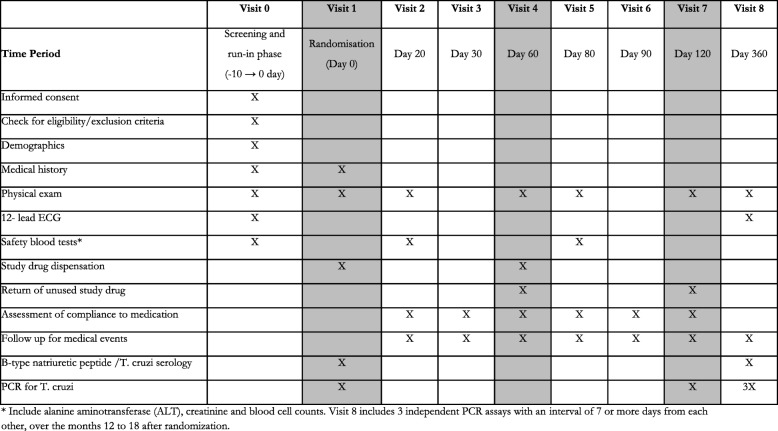
Overview of study visits and procedures

The visit schedule may vary at centres outside Colombia, according to the chosen study groups, the practice patterns and preferences. In all cases, however, the schedule should include in-person evaluations (also allowing for blood safety monitoring) at least every 30 days (i.e. 30, 60, 90 for those testing 120-day treatments), at end of treatment and 1 year after randomisation (for outcome assessment). Visit scheduling will adjust for centres testing only 60-day treatments. For those having 120HD treatments, it will be necessary to renovate the study treatment at 60 days in order to re-supply participants with the rest of the intervention and to test adherence. When applicable, these centres will decide whether or not to include a new follow-up visit to promote adherence for the next period and/or test safety (if so, including blood work). All centres must also commit to schedule additional visits at any other time during treatment upon a participant’s request.

### Laboratory testing

As presented in Table 2, some of the visits will include laboratory testing for treatment safety monitoring or study outcomes. The former includes blood cell counts and liver and kidney function tests, and the latter conventional *T. cruzi* serology and brain natriuretic peptides. Processing of blood safety monitoring tests will take place at referral laboratories for each centre as every participant attends his/her follow-up visits. Efficacy outcome data will have, in contrast, a centralised collective processing on stored samples in laboratories of reference once the follow-up of participants has finished. Participants will be individually informed of the results of these tests (the safety monitoring during the follow-up visits and the efficacy outcome when they are processed) and be given individualised copies of the laboratory reports.

Processing and interpretation for *T. cruzi* serology and BNP assays will follow recommendations of the makers of approved, commercially available assays. Techniques for PCR will follow consensus recommendations described elsewhere [16, 17]. Collection requires at least 3.5 mL of whole blood in tubes with ethylenediaminetetraacetic acid (EDTA), in triplicate. These samples are then mixed into equal parts with guanidine hydrochloride-6 M EDTA and stored at 4 °C (2–8 °C) until processing. Aliquots of 500 μL will be taken to conduct the extraction of DNA using the High Pure PCR Template Preparation Kit (Roche). Analysis of DNA samples using real-time PCR amplifies the repetitive region of DNA of the parasite with the initiators cruzi1 and cruzi2. For conventional PCR we will use the primers S21 and S22. The lab will conduct triplicate analyses of the blood samples, and the absolute quantification of the parasite will be the average of the replicates.

### Sample size calculations

The planned hypotheses in this study will be based on the following assumptions for its sample size:

1. The proportion of participants in the placebo group with at least one positive PCR out of three tests (1 + PCR, the primary outcome for efficacy) will be 75% or higher.
2. As a standard of trypanocidal efficacy, the proportion of (1 + PCR) in the group receiving conventional treatment with BZN (based on results of the BENEFIT trial) will be up to 30% (or 25% in a group receiving BZN in a regime with better tolerance).
3. A treatment with NFX, to be considered non-inferior to BZN, should have at least two thirds of its trypanocidal effect, which is, in terms of the above assumptions, 45% of 1 + PCR (or up to 50% in a group receiving a regime with lower tolerance/efficacy).

Based on the above assumptions, having at least 60–80 participants per study group will allow enough (at least 80%) power to identify a true difference between NFX and placebo or BZN (as negative or positive control for superiority or non-inferiority, respectively), testing a one-tailed hypothesis at an alpha level of 1%. Colombian centres should account for at least 60% of the study population, or 300 participants in the study (60 participants for each group). The overall goal of recruitment, including participants for centres outside Colombia, will be 500, as long as they include at least 80 participants allocated to every study arm (which may vary, depending on the treatment groups chosen at centres outside Colombia). Table 3 shows the scenarios for power calculations for the comparisons on efficacy.

**Table 3:**
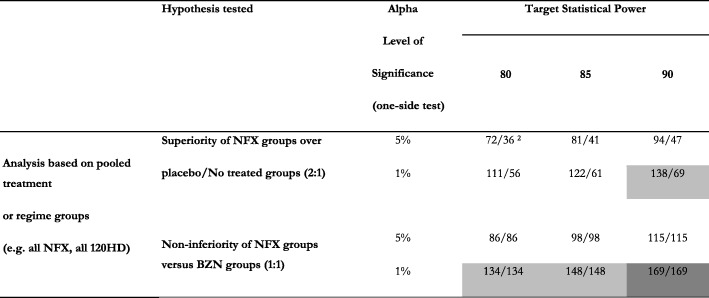
Sample size calculations for the primary comparisons in the study ^1^

^1.^ Needed for each comparison group, based on assumptions of trypanocidal effect explained in the text, including 5% of participant losses

^2.^ Cells with no shade imply feasible scenarios with 60 participants for each group, the recruitment goal for Colombia. *Light grey* cells imply the need to have 60–80 participants per group to make this comparison viable. *Dark grey* cells imply the need to have more than 80 participants per group

### Randomisation

A central, computerised, web-supported application will both assign the study treatment and support remote data collection for every participant. Study personnel at each centre will have their own stock of coded treatments. The system will assign a new treatment upon request at each centre, using random permutations within blocks for predefined strata of age (20–24 years, 25–40 years, 41/older), electrocardiogram status (abnormal /normal) and study centre. That way the randomisation sequence will be concealed for study personnel.

The system will adapt the randomisation scheme to each centre, according to their decision on which treatment groups to include. For Colombian centres, block sizes will be every 5 treatments (one for each study arm). For the rest of the centres (having no masked treatments) the system will assign 2, 3 or 5 different treatments (depending on the inclusion of 2 or 4 active treatment groups, and a control group with deferred treatment). The system will keep the same randomisation strata, but the block sizes will depend on the number of study groups chosen at each centre. In order to ensure concealment of allocation sequence, block sizes will randomly vary at these centres between the number of study groups and its double (i.e. 3 or 6 at a centre having 3 treatment arms).

For Colombian centres, which are offering 120-day-long, masked treatments, the system will allocate two consecutive sequences of 60 days. The groups allocated to active treatment for 60 days (60CD) will have a concealed, randomly allocated period of masked placebo (as treatment for days 1–60 or 61–120) and the remaining days with active medication.

### Data collection and overseeing

Data collected in the study visits and procedures will be entered into a central system, through standard case report forms (CRFs) available to authorised personnel. Most fields in the study CRFs include safeguards (i.e. fields allowing only numbers, a limit of characters or amounts entered). The quality of the information entered will be verified by centralised random validation (10%) and predefined crosschecks between fields of CRFs for each centre. The data will be stored in a secure and transportable system for storage and future analysis.

CRFs for centres outside Colombia may be adapted to local needs, including the visit schedule given by their choice of active treatment or the presence of untreated groups.

### Statistical analysis

Reporting of trial results will include a description of the enrolment process and the most relevant characteristics of the participants. For this purpose we will use counts and proportions or means and standard deviations for discrete and continuous variables, respectively.

Our outcome measures will be discrete variables, with the exemption of mean changes in the BNP levels, a secondary outcome for efficacy. For the purpose of evaluating the efficacy of NFX over placebo, we will test a hypothesis of no difference in the proportions of 1 + PCR at an alpha level of 1%. For the purpose of testing the equivalence of NFX to BZN or between 60CD and 120HD regimes, we will run a one-tailed non-inferiority test at an alpha level of 1%. In order to test for agent-dose interaction for the above comparisons, we will construct logistic regression models with the primary outcome as the event, and the comparison groups and study arms (e.g. NFX 60CD or NFX 120HD) as predictor variables. We will include the interaction dose*agent term in the model and run this test for no differences at an alpha level of 5%.

For the purpose of evaluating mean changes of BNP among groups, we will test for differences using independent-sample Student’s *t* tests comparing the groups of interest (alpha level of 1%). For the dose-agent interaction test (alpha level 5%), we will run an analysis of covariance with the mean changes as response, the comparison and study arms as factors and the interaction term.

## Discussion

Closing gaps in clinical research is critical for a field like Chagas disease, where major initiatives focused over the last decades on vector control to interrupt transmission [18, 19]. Most with relatively little data, particularly trials on trypanocidal treatment, have originated in Brazil and Argentina. More recently, there have been trials originating outside Latin America (e.g. in Spain and Canada). In the landscape of research on trypanocidal therapy, the EQUITY trial will tackle an important question on therapy for *T. cruzi* infection. As both NFX and BZN are currently used under recommendations and distribution of health authorities, this may be seen as a phase IV study. However, by expanding and validating the information on the effects of NFX, the study has the potential to challenge current recommendations on using alternatively NFX or BZN as similar interventions. In particular, this trial may benefit its target population from countries with fewer or no data from previous trials, or those where the best evidence indicates that BZN lacks a trypanocidal effect. Colombia, the country originating this trial, fits this situation. EQUITY thus represents a step forward for Colombia in terms of conducting this investigator-initiated, publicly funded trial for this largely neglected disease [20].

EQUITY will provide data and insight on three important aspects regarding the use of the two recognised possibilities for trypanocidal therapy, BZN and NFX. While testing the efficacy of NFX as a trypanocidal agent and the equivalence of these two agents, this trial will also test two different treatment regimes. The process to establish the daily dose and length of treatment for BZN and NFX (two agents developed since the 1960s) did not undergo rigorous evaluation. Recent reports on pharmacokinetics of BZN in children have suggested that under the recommended daily dose for adults (5 mg/kg a day) blood levels would exceed by twofold those with a proven trypanocidal effect for children [21]. If that is the case, and side effects are dose-related, halving the dose may promote tolerance. Conversely, another study in adults has suggested that serum levels of BZN are not associated with side effects [22]. Other authors have suggested that the length of exposure (i.e. cumulative doses) is what may trigger intolerance to BZN [23]. Others have, in fact, started tests of shorter, intermittent treatments with BZN [24]. Guidelines for treatment of *T. cruzi* infection indeed vary in the dose, but also in the length of therapy, mainly between 60 and 90 days [25]. Through comparison of the 60CD and 120HD treatment schemes, our study will inform whether tolerance to nitro derivatives NFX or BZN treatment is associated with doses or exposure.

EQUITY has some limitations. Firstly, its primary efficacy outcome is parasite-related. Establishing an effective regime that is well tolerated is an intermediate, necessary step for future testing of its clinical impact. In fact, in the placebo-controlled BENEFIT trial (where 20% of the population did not respond to BZN and 13% abandoned their treatment), the clinical impact went consistently towards benefit, but without statistically significant effect (7% reduction in its primary outcome, a composite of cardiac complications or death) [11]. Thus, choice of a treatment scheme with a better efficacy/tolerance ratio would be critical to identify a potential clinical impact. Secondly, as the trial relies on limited resources, it will have a limited geographical variety in its population. By having Colombia as the main participating country, we will address two needs: to run a new trial testing NFX (the first in Colombia, where a previous quasi-experiment showed positive results [26]), and to replicate (or challenge) previous results of the BENEFIT trial showing no trypanocidal effect. Thirdly, our study will test a limited amount of possibilities for trypanocidal therapy. The EQUITY trial will focus on the foundations to use NFX and to recommend it as an equivalent of BZN. Keeping the trial within this reach allows statistical power for the two main questions, at a cost of leaving, for example, combinations, other treatment schemes and other agents untested.

Finally, conducting a trial like EQUITY will, for the local capacity, provide a useful and constructive experience. This trial will increase much-needed clinical trial data on *T. cruzi*-infected populations from Andean countries. In that regard, EQUITY will join two recent trials including Bolivian patients or immigrants living in Spain [12, 13]. Investigator-initiated trials originating from Colombia are still scarce, and are a challenge for promotion among funding and regulatory agencies as well as research departments and laboratories. For this and other neglected tropical diseases, this type of trials may also be seen as a responsibility for endemic countries.

### Trial status

The Protocol Version is 2.0, dated 6 November 2015. The EQUITY trial obtained external funding (call 569, 2012) from Colciencias in February 2014. After obtaining institutional approvals, receiving raw study drugs from the ministry of health, re-packing the study medication and building the randomisation system, recruitment started on October 2015 in Colombia. Recruitment in additional countries started in June 2018 in Argentina. EQUITY is actively seeking up to three additional centres in southern cone countries. We will keep recruitment open outside Colombia up to having 500 participants or until the end of June 2019, whichever comes first. Last follow-up and database closure are expected by the end of 2020, and final results are planned to be reported by May 2021.

## Additional files

Additional file 1: SPIRIT 2013 checklist: recommended items to address in a clinical trial protocol and related documents. (DOC 120 kb)

Additional file 2: Ethics board approval, Fundación Oftalmológica de Santander (english version certificate). (PDF 479 kb)

Additional file 3: Funding Decision Certification. (PDF 26 kb)

## Data Availability

Any data and materials supporting information of this article can be requested from the corresponding author.

## Abbreviations

ALAT: Alanine amino transferase
ASAT: Aspartate amino transferase
BID: Twice a day
BZN: Benznidazole
CD: Conventional dose
EDTA: Ethylenediaminetetraacetic acid
HD: Half dose
NFX: Nifurtimox
PCR: Polymerase chain reaction
TC +: *T. cruzi* positive serology

## References

[1] World Health Organization. Chagas disease (American trypanosomiasis): epidemiology. WHO web site 2018. http://www.who.int/chagas/epidemiology/en/. Accessed 8 Oct 2018.

[2] Apt W, Heitmann I, Jercic MI, Jofré L, Muñoz P, Hauck IN (2018). Guía Clínica "Guías de Diagnóstico, Tratamiento y Prevención de la Enfermedad de Chagas".

[3] Equipo de trabajo Convenio 637/09 deCooperación OPS/OMS-MPS (2010). Guia de Atencion Clinica de la enfermedad de Chagas 2010.

[4] Unidad deEpidemiología PNdC (2007). Manual de Normas Técnicas y Operativas para el Tamizaje, Diagnóstico y Tratamiento de la Enfermedad de Chagas Crónica Reciente Infantil.

[5] Centers for Disease Control and Prevention. Parasites - American trypanosomiasis (also known as Chagas disease): antiparasitic treatment. CDC web site 2018 May 18. https://www.cdc.gov/parasites/chagas/health_professionals/tx.html. Accessed 9 Oct 2018.

[6] Coura JR, Borges-Pereira J (2011). Chronic phase of Chagas disease: why should it be treated? A comprehensive review. Mem Inst Oswaldo Cruz.

[7] Villar JC, Perez JG, Cortes OL, Riarte A, Pepper M, Marin-Neto JA (2014). Trypanocidal drugs for chronic asymptomatic Trypanosoma cruzi infection. Cochrane Database Syst Rev.

[8] de Andrade AL, Zicker F, de Oliveira RM, Almeida SS, Luquetti A, Travassos LR (1996). Randomised trial of efficacy of benznidazole in treatment of early Trypanosoma cruzi infection. Lancet.

[9] Sosa-Estani S, Segura EL, Ruiz AM, Velazquez E, Porcel BM, Yampotis C (1998). Efficacy of chemotherapy with benznidazole in children in the indeterminate phase of Chagas' disease. Am J Trop Med Hyg.

[10] Molina I, Prat J, Salvador F, Trevino B, Sulleiro E, Serre N (2014). Randomized trial of posaconazole and benznidazole for chronic Chagas' disease. N Engl J Med.

[11] Morillo CA, Marin-Neto JA, Avezum A, Sosa-Estani S, Rassi A, Rosas F (2015). Randomized trial of benznidazole for chronic Chagas' cardiomyopathy. N Engl J Med.

[12] Morillo CA, Waskin H, Sosa-Estani S, Del Carmen BM, Cuneo C, Milesi R (2017). Benznidazole and posaconazole in eliminating parasites in asymptomatic T. cruzi carriers: the STOP-CHAGAS trial. J Am Coll Cardiol.

[13] Torrico F, Gascon J, Ortiz L, Alonso-Vega C, Pinazo MJ, Schijman A (2018). Treatment of adult chronic indeterminate Chagas disease with benznidazole and three E1224 dosing regimens: a proof-of-concept, randomised, placebo-controlled trial. Lancet Infect Dis.

[14] Coura JR, de Abreu LL, Willcox HP, Petana W (1997). Comparative controlled study on the use of benznidazole, nifurtimox and placebo, in the chronic form of Chagas' disease, in a field area with interrupted transmission. I. Preliminary evaluation. Rev Soc Bras Med Trop.

[15] Yun O, Lima MA, Ellman T, Chambi W, Castillo S, Flevaud L (2009). Feasibility, drug safety, and effectiveness of etiological treatment programs for Chagas disease in Honduras, Guatemala, and Bolivia: 10-year experience of Medecins Sans Frontieres. PLOS Negl Trop Dis.

[16] Schijman AG, Bisio M, Orellana L, Sued M, Duffy T, Mejia Jaramillo AM (2011). International study to evaluate PCR methods for detection of Trypanosoma cruzi DNA in blood samples from Chagas disease patients. PLOS Negl Trop Dis.

[17] Ramirez JC, Cura CI, da Cruz MO, Lages-Silva E, Juiz N, Velazquez E (2015). Analytical validation of quantitative real-time PCR methods for quantification of Trypanosoma cruzi DNA in blood samples from Chagas disease patients. J Mol Diagn.

[18] Villar JC (2001). Commentary: Control of Chagas' disease: let's put people before vectors. Int J Epidemiol.

[19] Bello CR, Aceijas C, Alves PAB, Garelick H (2017). Evolution of Chagas' disease in Brazil. Epidemiological perspective and challenges for the future: a critical review. Perspect Public Health.

[20] Yamey G, Hotez P (2007). Neglected tropical diseases. BMJ.

[21] Altcheh J, Moscatelli G, Mastrantonio G, Moroni S, Giglio N, Marson ME (2014). Population pharmacokinetic study of benznidazole in pediatric Chagas disease suggests efficacy despite lower plasma concentrations than in adults. PLOS Negl Trop Dis.

[22] Pinazo MJ, Guerrero L, Posada E, Rodriguez E, Soy D, Gascon J (2013). Benznidazole-related adverse drug reactions and their relationship to serum drug concentrations in patients with chronic Chagas disease. Antimicrob Agents Chemother.

[23] Cancado JR (2002). Long term evaluation of etiological treatment of Chagas disease with benznidazole. Rev Inst Med Trop Sao Paulo.

[24] Alvarez MG, Hernandez Y, Bertocchi G, Fernandez M, Lococo B, Ramirez JC (2016). New scheme of intermittent benznidazole administration in patients chronically infected with Trypanosoma cruzi: a pilot short-term follow-up study with adult patients. Antimicrob Agents Chemother.

[25] Olivera MJ, Fory JA, Olivera AJ (2017). Therapeutic drug monitoring of benznidazole and nifurtimox: a systematic review and quality assessment of published clinical practice guidelines. Rev Soc Bras Med Trop.

[26] Bianchi F, Cucunuba Z, Guhl F, Gonzalez NL, Freilij H, Nicholls RS (2015). Follow-up of an asymptomatic Chagas disease population of children after treatment with nifurtimox (Lampit) in a sylvatic endemic transmission area of Colombia. PLOS Negl Trop Dis.

