# Correction to: Nifurtimox versus benznidazole or placebo for asymptomatic Trypanosoma cruzi infection (Equivalence of Usual Interventions for Trypanosomiasis - EQUITY): study protocol for a randomised controlled trial

**DOI:** 10.1186/s13063-019-3630-y

**Published:** 2019-08-20

**Authors:** Juan Carlos Villar, Víctor Mauricio Herrera, Juan Guillermo Pérez Carreño, Eliana Váquiro Herrera, Yeny Zulay Castellanos Domínguez, Skarlet Marcell Vásquez, Zulma Milena Cucunubá, Nilda Graciela Prado, Yolanda Hernández

**Affiliations:** 1 Grupo de Cardiología Preventiva, Facultad de Ciencias de la Salud, Universidad Autónoma de Bucaramanga, Calle 157 No 19- 55, Campus el, Bosque, Bucaramanga, Colombia; 2 Departamento de Investigaciones, Fundación Cardioinfantil- Instituto de Cardiología, Bogotá, Colombia; 3 Dirección de Investigación e Innovación, Universidad del Rosario, Bogotá, Colombia; 4 Grupo de Parasitología, Instituto Nacional de Salud, Bogotá, Colombia; 5 MRC Centre for Global Infectious Disease Analysis (MRC GIDA), Department of Infection Disease Epidemiology, Imperial College London, London, UK; 6 Departamento de Clínica, Patología y Tratamiento, Instituto Nacional de Parasitología Mario Fatala Chabén, Buenos Aires, Argentina

**Correction to: Trials**

**https://doi.org/10.1186/s13063-019-3423-3**

Following publication of the original article [1], the authors notified us of a few requested editions that were not implemented adequately during proofing. The publisher apologizes for the inconvenience caused to our authors and readers.

- page 3, paragraph 3 under the “Interventions” section, the reference to Table 1 was deleted from the text
- page 4, first paragraph under the “Comparisons” section, BZN was replaced with BZN/60CD and NFX was replaced with NFX/120HD
- page 5, the reference to Figure 2 was replaced with Table 2 within the text
- page 6, first paragraph, 1st line, under the “Laboratory testing” section, “Outcome date” should read “Efficacy outcome data” and “the outcome when they are processed” on the 6th line should read “the efficacy outcome when they are processed”
- page 7:
  - the reference to Table 2 was replaced with Table 3
  - first paragraph, 4th line, under the “Randomisation” section, the text “of similar aspect, whose content is unknown” was deleted
  - first paragraph, 4th line, under the “Discussion” section, “Most of these trials with relatively little data” should read “Most with relatively little data”
- In both figures of the manuscript, referring to the study drugs, the short forms used for Benznidazole (BZD) were different from what is used throughout the manuscript (BZN). The BZN for is kept.
- The figure and table captions have been corrected:
  Figure 2
  - Incorrect caption: Process of treatment allocation
  - Corrected caption: Daily 60-day treatment schedule for study participants
  Table 2
  - Incorrect caption: Sample size calculations for the primary comparisons in the study ^1^
  - Corrected caption: Overview of study visits and procedures
- Table 3 was added to the manuscript
- The name of Additional file 2 was changed from “The undersigned president of the ethics board for clinical research at the fundacion oftalmologica de santander foscal” to “Ethics board approval, Fundación Oftalmológica de Santander (english version certificate)”
- The acknowledgment for Nicolas’ Gelvez contribution to the manuscript was moved to the end of the “Acknowlegments” section

The original article has been corrected.
