# Supplementary material for: Nifurtimox versus benznidazole or placebo for asymptomatic Trypanosoma cruzi infection (Equivalence of Usual Interventions for Trypanosomiasis - EQUITY): study protocol for a randomised controlled trial
Source: Trials. 2019 Jul 15;20:431. doi: 10.1186/s13063-019-3423-3 (PMC6631895; doi:10.1186/s13063-019-3423-3)
Supplement: Supplementary file 2 — Ethics board approval, Fundación Oftalmológica de Santander (english version certificate). (PDF 479 kb) [file 13063_2019_3423_MOESM2_ESM.pdf]

THE UNDERSIGNED PRESIDENT OF THE ETHICS BOARD FOR CLINICAL RESEARCH AT THE FUNDACION OFTALMOLOGICA DE SANTANDER FOSCAL

certifies that:

The board (***Comité de Ética en Investigación de la Fundación Oftalmologica de Santander CEI- FOSCAL***) reviewed, discussed and approved the research protocol: "Cardiovascular Health Investigation and Collaboration from Countries of America to Assess the Markers and Outcomes of Chagas disease (CHICAMOCHA 3) – EQUITY (Equivalence of Usual Interventions for Trypanosomiasis". The approval was given and registered in the minutes of the board meeting on November 21rst, 2014.

This certificate is issued on October 24, 2018 for the purpose of submitting a manuscript to an international medical journal.

CARLOS PAREDES GOMEZ
Board President CEI- FOSCAL
