# Supplementary material for: Nifurtimox versus benznidazole or placebo for asymptomatic Trypanosoma cruzi infection (Equivalence of Usual Interventions for Trypanosomiasis - EQUITY): study protocol for a randomised controlled trial
Source: Trials. 2019 Jul 15;20:431. doi: 10.1186/s13063-019-3423-3 (PMC6631895; doi:10.1186/s13063-019-3423-3)
Supplement: Supplementary file 3 — Funding Decision Certification. (PDF 26 kb) [file 13063_2019_3423_MOESM3_ESM.pdf]

El conocimiento es de todos | Colciencias

Bogotá, January 17, 2019

The Editorial Office

Trials Journal

Ref: Funding Decision Certification

Dear Sir/Madam:

As the head of the National Program in Health Sciences Research and Technology at Colciencias, the Colombian government granting agency, I certify that the project "Cardiovascular Health Investigation from Countries of America to Assess the Markers and Outcomes of Chagas Disease (CHICAMOCHA 3) – Equity (Equivalence of Usual Interventions for Trypanosomiasis" received funding through the contract 729-2013, code 124156935014.

Our funding decisions come from annual granting competitions led by our agency branch. Every year Colciencias receives 400-500 proposals from researchers across the country, dealing with the topics of interest addressed in a single call. After an initial review for administrative requirements, all eligible proposals undergo an independent and plural peer review process. Depending on the quality of the proposals and the availability of funding for each of our calls, 50-70 of those proposals are awarded every year.

The CHICAMOCHA 3 – EQUITY project was declared eligible for funding in the 2013 annual competition. The contract started in December, 2013, and lasted 54 months. The amount awarded in the contract was $327.483.279 (roughly 170.000 USD at the time). This amount covered 56% of the cost of the research activities included in the grant proposal. It is our policy that research contracts include both financial support from both Colciencias (up to 70% of the proposed activities) and the institution(s) carrying out the project. Colciencias found no technical or financial issues in the execution of this contract.

I am giving this certificate upon request from Dr. Villar, principal investigator of this project for the submission of the study protocol to the Trials Journal. Please do not hesitate to contact me, should you require any additional detail or information in this matter.

Sincerely,

Diana M Calderon, PhD

Head of the National Program in Health Sciences Research and Technology

• **Centro de contacto:**

PBX: (57+1) 6258480, Ext. 2081
Línea gratuita nacional: 018000914446
Código postal: 111321

Av. Calle 26 Nº 57-83
Torre 8, Piso 2 al 6
Bogotá D.C. Colombia
